# Factors associated with the incomplete opportune vaccination schedule up to 12 months of age, Rondonópolis, Mato Grosso

**DOI:** 10.1590/1984-0462/2022/40/2020300

**Published:** 2021-11-12

**Authors:** Patrícia de Lima Lemos, Gilmar Jorge de Oliveira, Nidyanara Francine Castanheira de Souza, Izadora Martins da Silva, Izabella Paes Gonçalves de Paula, Karoline Cordeiro Silva, Fernanda Camargo Costa, Poliana Duarte da Silva Arruda, Washington Júnior Oliveira, Poãn Trumai Kaiabi, Michelli Clarisse Alves Passarelli, Amanda Cristina de Souza Andrade, Olga Akiko Takano

**Affiliations:** a Universidade Federal de Mato Grosso, Cuiabá, MT, Brazil.; b Universidade Federal de Mato Grosso, Rondonópolis, MT, Brazil.

**Keywords:** Health survey, Vaccination, Vaccination coverage, Children, Inquérito epidemiológico, Vacinação, Cobertura vacinal, Crianças

## Abstract

**Objective:**

To analyze factors associated with the incomplete timely vaccination schedule up to 12 months of age, in children born in 2015, in the municipality of Rondonópolis, Mato Grosso.

**Methods:**

Population survey, August/2017 to February/2018, which used the method proposed by the World Health Organization to collect information about routine vaccination. For analysis of the associated factors, the recommendations of the National Immunization Program of the Ministry of Health were considered. Univariate analysis was performed, and the factors associated with p<0.20 entered in the multiple analysis, with hierarchical entry of individual variables and contextual indicator of concentration of the income extremes.

**Results:**

The incomplete timely vaccination schedule up to 12 months was 82.03% (95%CI 78.41–86.63). In the final model, the following remained independently associated: having one or more siblings at home (OR 3.18; 95%CI 1.75–5.76) and not receiving a visit from a community health worker in the last 30 days (OR 1.93; 95%CI 1.04–3.57).

**Conclusions:**

It is necessary to implement an active search for children with vaccination delay in relation to the recommended interval for each vaccine, in addition to the need to strengthen the link of the family health strategy and child caregivers.

## RESUMO

**Objetivo:**

Analisar fatores associados ao esquema vacinal oportuno incompleto até os 12 meses de idade, em crianças nascidas em 2015, no município de Rondonópolis, Mato Grosso.

**Métodos:**

Inquérito populacional, agosto/2017 a fevereiro/2018, que utilizou o método proposto pela Organização Mundial da Saúde para coletar informações sobre a vacinação de rotina. Para analisar os fatores associados, consideraram-se as recomendações do Programa Nacional de Imunização do Ministério da Saúde. Realizou-se análise univariada e os fatores associados com p<0,20 entraram na análise múltipla, com entrada hierarquizada das variáveis individuais e indicador contextual de concentração de extremos de renda.

**Resultados:**

O esquema vacinal oportuno incompleto até os 12 meses foi de 82,03% (IC95% 78,41–86,63). No modelo final, permaneceram independentemente associados: possuir um irmão ou mais no domicílio (OR 3,18; IC95% 1,75–5,76) e não receber visita de agente comunitário de saúde nos últimos 30 dias (OR 1,93; IC95% 1,04–3,57).

**Conclusões:**

É necessário implementar busca ativa de crianças com atraso vacinal em relação ao intervalo recomendado para cada vacina, além da necessidade de fortalecer o vínculo da estratégia de saúde da família e cuidadores de crianças.

## INTRODUCTION

Immunization is considered one of the main measures of disease prevention in public health, a priority for surveillance and primary health care. Throughout its 45 years of history, the National Immunization Program (PNI) of the Ministry of Health (MS) of Brazil has records of great achievements with regard to the control, reduction and/or elimination of vaccine-preventable diseases.^1^ The PNI/MS acts in line with the Global Vaccination Action Plan of the World Health Organization (WHO), developed in 2012, aimed at improving access to vaccination worldwide and preventing deaths by 2020.^1,2^

Currently, 12 vaccines for 19 diseases are available on the child's calendar. The increase in the complexity of the vaccination schedule, especially for children, culminated in new challenges, such as reaching and maintaining vaccination coverage (VC) in the targets established by the PNI/MS.^1,3,4^

Despite the advances and high VC achieved since 1990, starting in 2016, there was a reduction in vaccination rates in the country, below the goals recommended by the PNI/MS, associated with an upsurge of already controlled diseases, such as measles, whooping cough and yellow fever.^3–5^

Several factors may be associated with a drop in VC, such as socioeconomic and demographic factors, low maternal education, worse living conditions, size of the family, older age and birth order of the child, outpatient care, shortage of vaccines, fake news (false news), lack of risk perception for diseases, and access to the vaccination service, among others.^6–12^

Thus, the objective of this study was to analyze factors associated with the incomplete timely vaccination schedule up to 12 months of age, in children born in 2015.

## METHOD

A population-based survey that used the cluster sampling method proposed by WHO to estimate VC, was conducted in Rondonópolis, Mato Grosso, August 2017 to February 2018.^13^

Rondonópolis is located in the Central-West region of Brazil. According to the 2010 Census, it has 195,476 inhabitants, a human development index (HDI) of 0.755 and a Gini index of 0.52.^14^

The study population consisted of the cohort of live births in 2015, which corresponded to 4,022 live births. For this study, the inclusion criteria were children born in Rondonópolis during 2015, with birth weight ≥2500 g, minimum of 20 months of age and vaccination booklet.^15^

To calculate the sample size, an expected VC of 50% was adopted, a significance level of 0.05, precision of the estimate of 0.10, effect of the design of 2, which resulted in 210 individuals; to increase the power of estimates, it was decided to double this number.^13^

For this study, the cluster was defined as a neighborhood, rather than a census sector, as there was more precise territorial delimitation in the digitized maps of the municipality.

**Figure 1:**
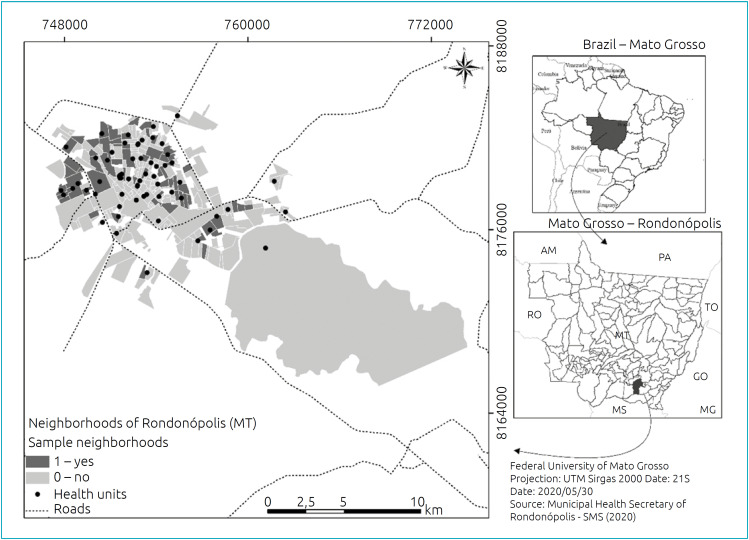
Spatial distribution of the neighborhoods selected for the household survey by health district, Rondonópolis, Mato Grosso, 2017 (n=60).

Stratified probability sampling was adopted according to the health district (north, south, east, west and central-west) and clusters following three stages: neighborhood, home and child. The municipality had 260 neighborhoods, and of these, 60 were drawn, proportionally to the number of children under 1 year of age in the five health districts, according to the 2010 Census data estimate (Figure 1).

The neighborhoods were then mapped, and a new random drawing was made to define the starting points of each street in the neighborhoods. In the collection, the interviewer visited the home and proceeded to the right side, by convention, across the street until reaching seven children per neighborhood, whenever possible one per street, belonging to the birth cohort of the year 2015. In households where there was more than one child in this cohort, the oldest was selected.

A total of 6,508 households were listed in a field diary, of which 532 were visited during the survey. There were 32 refusals and 60 losses due to the absence of the parents/guardians and/or the vaccination booklet after three visits, totaling 434 children selected for this study.

The team of interviewers consisted of seven medical students and two nursing students. Training sessions were held for data collection, standardization of instruments, photography of the vaccination booklet and pilot test. The data obtained by the pilot test were not included in the analysis.

To collect the data, parents/guardians were interviewed with a questionnaire containing socioeconomic, demographic and health services-related information. Data from the Live Birth Information System (SINASC) and the Brazilian Institute of Geography and Statistics (IBGE) were used.^14^

To analyze the vaccination of the children in this study, the vaccination schedule established by the PNI/MS in 2015 was considered:^16^

- **At birth:** Bacillus Calmette-Guérin (BCG) and hepatitis B (HB).
- **2 months:** Penta1 (diphtheria, tetanus, pertussis, hepatitis B, *Haemophilus influenzae* b), IPV1 (inactivated polio vaccine), Pneumo10-1 (10-valent pneumococcal conjugate vaccine) and Rota1 (oral human rotavírus vaccine).
- **3 months:** MeningoC1 (meningococcal C conjugate).
- **4 months:** Penta2 (diphtheria, tetanus, pertussis, hepatitis B, *Haemophilus influenzae* b), IPV2 (inactivated polio vaccine), Pneumo10-2 (10-valent pneumococcal conjugate vaccine) and Rota2 (oral human rotavírus vaccine).
- **5 months:** MeningoC2 (meningocócica C conjugada).
- **6 months:** Penta3 (diphtheria, tetanus, pertussis, hepatitis B, *Haemophilus influenzae* b), OPV3 (oral polio vaccine).
- **9 months:** yellow fever (YF).
- **12 months:** Pneumo10-R, MMR (measles, mumps, rubella, 1st dose), hepatitis A (HA).
- **15 months:** DTP (diphtheria, tetanus and pertússis, booster), OPV-R (booster), Meningo C-B (booster) and MMR-C (measles, mumps, rubella and chickenpox, 2nd dose MMR and/or single dose chickenpox or tetra viral).

The **outcome variable** was an incomplete timely vaccination schedule up to 12 months of age (no; yes). An incomplete schedule was considered not having received any of the doses previously listed, at the age and in the interval established by the PNI/MS. A delayed dose was understood as the vaccine applied at 30 days or more of the recommended age.

HA and Rota vaccines were not considered to analyze the vaccination schedule until 12 months: the first was implanted in the basic calendar in the year of the birth cohort of this study, the second, for having a rigid interval, was analyzed separately.

The index of concentration at the extremes (ICE) for income was calculated, which is an indicator of socio-spatial segregation used to determine how people are economically and socially concentrated, that is, it is a measure of social polarization that considers extremes superior and inferior at the contextual level.^17^

ICE was calculated using the following formula: ICE_i_=(A_i_ - P_i_)/T_i_, with A_i_ being the number of least privileged people in a given geo graphical area, P_i_ the number of most privileged people and T_i_ the total population. The indicator ranges from -1 to 1, with a value of 1 indicating that 100% of the population is at the most privileged level.^17^

The ICE calculation considered the per capita household income variable only for the selected neighborhoods (n=60) in this study. Four neighborhoods (total of 31 children in the sample) that did not exist in the IBGE 2010 Census database were excluded. The cutoff points for the extremes of per capita household income were defined by the 20th and 80th percentiles. Less privileged people were those whose per capita household income was less than or equal to half the minimum wage (A_i_), and those with household income were privileged. per capita greater than or equal to two minimum wages (P_i_), divided by the total population of the neighborhoods (T_i_).^14,17^

For analysis, ICE was converted to a binary variable, whose categories were called deprivation (negative values) and privilege (positive values).

**Figure 2:**
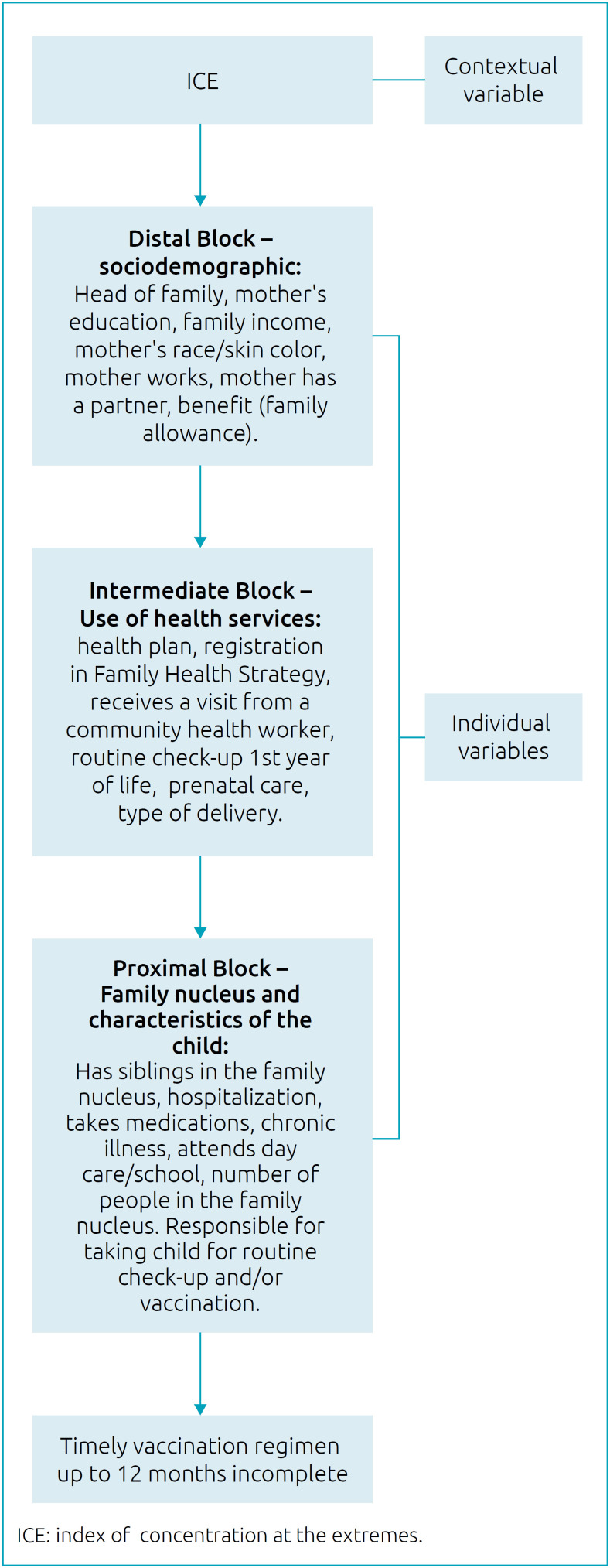
Hierarchical entry per blocks of variables, to analyze the factors associated with incomplete timely vaccination schedule up to 12 months of age Rondonópolis, Mato Grosso, 2017.

The **independent variables** were organized in hierarchical blocks, according to the consulted literature (Figure 2): socioeconomic and demographic context, use of health services, constitution of the family nucleus and characteristics of the child, according to the consulted literature.^6–12,18^

The prevalence and 95% confidence interval (95%CI) of the incomplete timely vaccination schedule up to 12 months were estimated, in addition to the estimate for each vaccine. To verify the factors associated with the incomplete vaccination schedule, odds ratio (OR) was estimated through binary logistic regression analysis using the generalized estimating equations (GEE) method, which considered the neighborhood as a cluster variable.

All variables with p<0.20 in the univariate analysis were maintained in the multiple model, and their entry in the model followed the order of the blocks (Figure 2).

First, the contextual variable (ICE) was included, followed by the sociodemographic variables (distal block), then the variables of use of health services (intermediate block) and those related to the family nucleus and child (proximal block). The variables of the distal blocks remained adjusted for the lower blocks.

The questionnaires were double entered in a digital spreadsheet and, to verify the consistency of the data, the data compare function of the Epi-Info program, version 3.5.4 was used. The analyses were performed using the statistical program Stata version 12.0 (StataCorp LP, College Station, United States), with a significance level of 5%.

The research was approved by the Research Ethics Committee of Hospital Universitário Júlio Müller, Assessment No. 1.878.72716 of December 16, 2016, CAAE No. 47227115.2.0000.5541. Only children whose parents/guardians agreed to participate and signed an informed consent form were included in the study.

## RESULTS

The incomplete timely vaccination schedule up to 12 months in 434 children was 82.03% (95%CI 78.41–85.63). The vaccines with the lowest values for the incomplete schedule were BCG (8.76%; 95%CI 6.10–11.41) and HB (4.61%; 95%CI 2.63–6.58). As for vaccines with two or three dose sequential schedules, the third dose of Penta (47.70%; 95%CI 42.99–52.39) was the most incomplete. The third dose of IPV/OPV and Penta, both applied at 6 months, showed different percentages (Table 1).

**Table 1:** Incomplete timely vaccination schedule up to 12 months of age and per vaccine for children born in 2015, Rondonópolis, Mato Grosso, 2017.

Vaccine		%	95%CI
BCG	Single dose	8.76	6.10–11.41
HB	Initial dose	4.61	2.63–6.58
Penta	1st dose	12.01	8.92–15.03
	2nd dose	32.49	28.08–36.89
	3rd dose	47.70	42.99–52.39
IPV/OPV	1st dose	11.98	8.92–15.03
	2nd dose	31.11	26.75–35.46
	3rd dose	46.54	41.85–51.23
Pneumo10	1st dose	16.13	12.66–19.58
	2nd dose	38.48	33.90–43.05
MeningoC	1st dose	20.97	17.13–24.79
	2nd dose	38.94	34.35–43.52
YF	Initial dose	50.92	46.21–55.62
MMR	1st dose	55.07	50.38–59.74
Incomplete schedule up to 12 months	–	**82.03**	**78.41–85.63**

95%CI: 95% confidence interval; BCG: bacillus Calmette-Guérin, tuberculosis vaccine; HB: hepatitis B vaccine; Penta: vaccine against diphtheria, tetanus, pertussis (whooping cough), *Haemophilus influenzae* b and hepatitis B; IPV/OPV: inactivated polio vaccine/oral polio vaccine; MeningoC: vaccine against meningococcal disease C (conjugated); Pneumo10: vaccine against pneumococcal disease 10 (conjugated); YF: yellow fever vaccine (attenuated); MMR: measles, mumps and rubella vaccine; the schedule was considered incomplete up to 12 months if none of the following doses were received in the correct interval: 1 dose BCG; 3 doses Penta; 3 doses IPV/OPV; 2 doses Pneumo10; 2 doses MeningoC; 1 dose YF; 1 dose MMR.

The ICE found was 79.90% in children who lived in less privileged neighborhoods. Most of the children were female (52.53%) and had an average age of 27 months (minimum of 20 and maximum of 36 months). In the variable “parents” as head of the family, 12.90% were women (mother) and 65.40% were men (father).

The main maternal characteristics were: schooling between 9 and 11 years of study, family income between one and three minimum wages, race/brown color, did not work outside the home and had a partner (Table 2).

**Table 2:** Univariate analysis of income index of concentration at extremes, socioeconomic and demographic characteristics, according to incomplete timely vaccination schedule up to 12 months, Rondonópolis, Mato Grosso, 2017 (n=434).

		N (%)	Incomplete schedule# (%)	OR∞(95%CI)
ICE€				
	Privilege	322 (79.90)	81.68	1.09 (0.59–2.03)
	Deprivation	81 (20.10)	80.25	1.00
Distal block –socioeconomic and demographic characteristics				
Head of familyᵃ				
	Others	94 (21.71)	85.11	1.09 (0.61–1.95)
	Fathers	339 (78.29)	81.12	1.00
Mother's education in years^b^				
	0-8	89 (20.70)	87.64	1.73 (0.85–3.52)
	≥9	341 (79.30)	80.94	1.00
Mother's age in years				
	≥20	398 (91.71)	83.42	**2.63 (1.25–5.55)**
	<20	36 (8.29)	66.67	**1.00**
Family income in minimum salaries¥ ^c^				
	<3	271 (63.17)	80.81	0.80 (0.47–1.36)
	≥3	158 (36.83)	84.81	1.00
Race/skin color of child reported by parents/guardian				
	White	162 (37.33)	82.72	1.36 (0.59–3.12)
	Brown	225 (51.84)	82.22	1.26 (0.57–2.79)
	Black	47 (10.83)	78.72	1.00
Race/skin color of mother self-reporte^d^d				
	White	118 (28.71)	83.90	1.53 (0.71–3.29)
	Brown	223 (54.26)	82.06	1.32 (0.67–2.59)
	Black	70 (17.03)	78.57	1.00
Mother works^e^				
	Yes	176 (40.74)	82.39	1.07 (0.64–1.80)
	No	256 (59.26)	81.64	1.00
Mother has partner^f^				
	No	86 (19.95)	79.07	0.69 (0.38–1.26)
	Yes	345 (80.05)	82.61	1.00
Beneficiary of the cash transfer program (Bolsa Família)				
	Yes	117 (26.96)	85.47	1.46 (0.80–2.66)
	No	317 (73.04)	80.76	1.00

# VC: vaccination coverage

∞ OR: crude odds ratio

¥ 95%CI: 95% confidence interval; ^¥^minimum salary in 2015=R$ 937.00

€ ICE: index of concentration at the extremes. Number of missing data (did not know/did not answer): a=1; b=4; c=5; d=23; e=2; f=3.

Regarding health services, 75.75% did not have a health plan and 37.10% did not receive a visit from a community health worker (CHW) in the last 30 days. In the family unit, 73.96% had siblings, 69.59% did not attend day care/school, and 78.57% of the children were under the responsibility of their parents to go to the routine check-up and/or vaccination (Table 2).

In the univariate analysis, a higher prevalence of incomplete timely vaccination schedule up to 12 months was observed among mothers aged 20 years or older, less than six prenatal consultations, children who had one or more siblings and whose home was not visited by a CHW in the last 30 days (Table 3).

**Table 3:** Univariate analysis of index of concentration at the extremes, use of health services, family nucleus and characteristics of child, according to incomplete timely vaccination schedule up to 12 months. Rondonópolis. Mato Grosso. 2017 (n=403).

		n (%)	Incomplete schedule# (%)	OR∞(95%CI)
Intermediate block– use of health services				
Health planᵃ				
	No	328 (75.75)	82.93	1.28 (0.72–2.25)
	Yes	105 (24.25)	80.00	1.00
Registered in PSF				
	No	70 (16.13)	77.14	0.65 (0.34–1.23)
	Yes	364 (83.87)	82.97	1.00
Visit from CHW				
	No	161 (37.10)	86.96	**1.93 (1.09–3.40)**
	Yes	273 (62.90)	79.12	1.00
Prenatal check-up				
	<6	132 (30.41)	86.36	**1.82 (1.00–3.30)**
	≥6	302 (69.59)	80.13	1.00
Type of delivery				
	Cesarean	250 (58.41)	82.00	1.13 (0.68–1.87)
	Normal	178 (41.59)	81.46	1.00
Routine check-up 1st year^c^				
	No	24 (5.58)	80.00	0.90 (0.29–2.77)
	Yes	406 (94.42)	81.50	1.00
Routine check-up 2nd year				
	No	80 (18.43)	81.30	0.84 (0.44–1.58)
	Yes	354 (81.57)	82.20	1.00
Proximal block – family nucleus and characteristics of child				
Has siblings				
	One or more	321 (73.96)	87.23	**3.88 (2.29–6.55)**
	None	113 (26.04)	67.26	1.00
Hospitalization				
	Yes	99 (22.81)	85.86	1.29 (0.68–2.44)
	No	335 (77.19)	80.90	1.00
Takes medications				
	Yes	30 (6.91)	80.00	0.91 (0.35–2.32)
	No	404 (93.09)	82.18	1.00
Has chronic disease				
	Yes	40 (9.22)	80.00	0.84 (0.37–1.92)
	No	394 (90.78)	82.23	1.00
Attends day care/school				
	Yes	132 (30.41)	84.85	1.28 (0.73–2.25)
	No	302 (69.59)	80.79	1.00
Number of people in household				
	>5	72 (16.59)	81.94	0.92 (0.47–1.80)
	≤5	362 (83.41)	82.04	1.00
Responsible for taking child for routine check-up and/or vaccination				
	Others	93 (21.43)	80.65	0.99 (0.53–1.84)
	Fathers	341 (78.57)	82.40	1.00

# VC: vaccination coverage

∞ OR: crude odds ratio

¥ 95%CI: 95% confidence interval; ^¥^minimum salary in 2015=R$ 937.00

€ ICE: index of concentration at the extremes; CHW: community health worker; PSF: Family Health Program; number of missing data (did not know/did not answer): a=1; b=4; c=5; d=23; e=2; f=3.

In the multiple analysis, the incomplete timely vaccination schedule remained independently associated with children who had one or more siblings and had not received a CHW visit for more than 30 days (Table 4).

**Table 4:** Multiple analysis of hierarchical blocks and incomplete timely vaccination schedule up to 12 months according to index of concentration at the extremes, Rondonópolis, Mato Grosso, 2017 (n=403).

		Incomplete timely vaccination schedule		
		Model 1 OR¥ (95%CI)#	Model 2 OR (95%CI)	Model 3 OR (95%CI)
ICE				
	Deprivation	1.21 (0.68–2.14)	1.23 (0.67–2.26)	1.22 (0.66–2.23)
	Privilege	1.00	1.00	1.00
Distal block–socioeconomic and demographic characteristics				
Mother's education in years				
	0-8	2.23 (1.04–4.77)	2.02 (0.92–4.43)	1.76 (0.78–3.97)
	≥9	1.00	1.00	1.00
Mother's age in years				
	≥20	2.94 (0.21–6.67)	3.23 (1.35–7.69)	1.79 (0.71–4.54)
	<20	1.00	1.00	1.00
Family income in minimum salaries				
	<3	0.78 (0.43–1.41)	0.75 (0.41–1.39)	0.69 (0.37–1.28)
	≥3	1.00	1.00	1.00
Mother works				
	Yes	0.96 (0.53–1.72)	0.95 (0.52–1.72)	0.84 (0.46–1.54)
	No	1.00	1.00	1.00
Mother has partner				
	No	0.64 (0.33–1.22)	0.65 (0.34–1.26)	0.60 (0.30–1.17)
	Yes	1.00	1.00	1.00
Intermediate block – health services				
Health plan				
	No	–	1.25 (0.67–2.31)	1.25 (0.67–2.35)
	Yes	–	1.00	1.00
Visit from community health worker in last 30 days				
	No	–	**1.97 (1.07–3.60)**	**1.93 (1.04–3.57)**
	Yes	–	1.00	1.00
Prenatal check-up				
	<6	–	1.73 (0.92–3.24)	1.56 (0.83–2.95)
	≥6	–	1.00	1.00
Proximal block – family nucleus and characteristics of child				
Has siblings in family nucleus				
	One or more	–	–	**3.18 (1.75–5.76)**
	None	–	–	1.00
Responsible for taking child for routine check-up and/or vaccination				
	Others	–	–	1.34 (0.66;2.71)
	Fathers	–	–	1.00

¥ OR: adjusted odds ratio

# 95%CI: 95% confidence interval.

**Figure 3:**
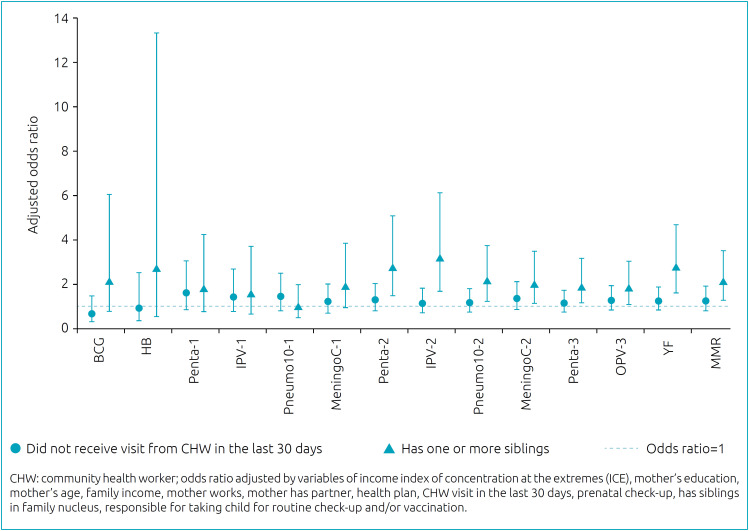
Association between each vaccine recommended in the basic vaccination schedule of the National Immunization Program of the Ministry of Health, according to the variables having one or more siblings and not receiving a visit from a community health worker in the last 30 days, Rondonópolis, Mato Grosso, 2017.

Incomplete vaccine/dose schedules were significantly associated with having one or more siblings at home for Penta 2 (OR 2.75; 95%CI 1.49–5.08), IPV2 (OR 3.21; 95%CI 1.69–6.11), Pneumo2 (OR 2.16; 95%CI 1.24–3.75), MeningoC2 (OR 2.01; 95%CI 1.15–3.49), Penta3 (OR 1.89; 95%CI 1.13–3.17), OPV3 (OR 1.82; 95%CI 1.08–3.04), YF (OR 2.77; 95%CI 1.64–4.68) and MMR (OR 2.10; 95%CI 1.25–3.50), and not having received a CHW visit for more than 30 days did not show any significant association between the vaccines analyzed alone (Figure 3).

## DISCUSSION

This study found a high prevalence of incomplete timely vaccination schedule up to 12 months and was higher among children who lived with one or more siblings and in households that did not receive a CHW visit in the last 30 days.

The incomplete timely vaccination schedule in this study (82.03%) was superior to that found in São Luís, Maranhão, where 23.60% of the children were not adequately vaccinated at 12 months of age.^12^ The vaccination survey carried out in the 27 Brazilian capitals, 2007–2008, with 17,749 children, showed that 18.20% of these were delayed with one or more vaccines recommended up to 18 months of age, but considered as an applied dose criterion.^11^

In Quebec, Canada, an analysis carried out from 2006 to 2016, including 7,183 children, showed that 23.60% of children had delayed vaccinations at 12 months of age and, in analysis at 24 months, 72.50% of children had an incomplete calendar, revealing progressive delays at 2 (5.42%), 4 (13.35%) and 6 (23.16%) months of age.^9^

Most VC studies analyze by dose applied according to the population, which makes it difficult to make comparisons with this study, and in addition, each country takes into account different vaccines and schedules up to 12 months of age.

A study in Delhi, India selected 458 migrant families divided into two groups, recent and settled, and investigated access to health services and the determinants of timely VC in children at 12 months of age. The incomplete vaccination schedule at 12 months among recent migrants was higher (69.00%) compared to settlers (47.00%).^19^

In this study, incomplete vaccination for BCG was different from HB and for Penta and IPV/OPV, although their simultaneous application is recommended; perhaps this reflects the shortage of one of these vaccines or the mother's fear of her child receiving more than one vaccine injection. Another factor is the need to return to the health service; for example, the BCG vaccine bottle is multi-dose, which is why each health unit defines a day of the week for its application. Other authors have also demonstrated low incomplete vaccination for BCG and HB.^10–12,20^

In this study, vaccines with multi-dose schedules showed a decrease in the percentage of vaccination at subsequent doses, similar to that found by other authors. In vaccines for diseases that require more than one dose, there is a greater delay in vaccination in subsequent doses, which requires strategies to improve adherence.^9,11,12^

Two points deserve to be highlighted regarding the importance of adhering to the child's basic vaccination schedule. The immune response is better when applied at the recommended age and when the minimum interval is respected.^21,22^ Another factor to be considered is the importance of timely vaccination, especially to prevent meningitis, pneumonia and pertussis in young infants.

In the multiple analysis, variables related to the intermediate block (use of health services), “children whose home did not receive a CHA visit in the last 30 days” and to the proximal block (family nucleus and child), “those who lived with a sibling or more”, remained independently associated with the incomplete timely vaccination schedule up to 12 months of age.

Regarding the CHW visit, a study carried out in India emphasized the importance of strengthening home visits by health professionals, a predictive factor for complete vaccination, especially in the postpartum period, when communication about the importance of childhood immunization can be more effective.^19^ A review study on interventions to raise VC revealed that in low- and middle-income countries, the information provided to parents and/or guardians during home visits represents an important predictor of complete vaccination.^23^

The finding of the CHW visit variable in the last 30 days may indicate that home visits are of paramount importance to timely complete the vaccination calendar and indicate the irregularity of home visits in the first year of life. According to the National Primary Care Policy (PNAB), home visits by CHWs must take place monthly or according to the condition of the family's vulnerability.^24^

The vaccination delay during the first year of life characterizes a situation of vulnerability, and its detection can occur during home visits or in the monthly verification of the vaccination booklet of all children assigned to the health unit.

As for the number of siblings, studies have shown that the greater number of children in the household implies a greater incomplete vaccination schedule, which can be explained by less time available to the mother, demand for more financial resources, family dynamics and less access to health services for vaccination.^9,10^ Contrary to the present study, in Pakistan, a larger number of children in the household was associated with complete vaccination.^25^

With regard to the number of siblings, improving the link between the health service and the enrolled population is essential to increase adherence to vaccination, especially in households with a greater number of children.

No differences were found between the contextual variable, the concentration index at the extremes and the incomplete timely vaccination schedule. In this study, the individual socioeconomic and demographic variables did not explain the incomplete timely vaccination schedule at 12 months of age.

Unlike the result of the contextual variable in this study, a study carried out in São Luís, Maranhão, using another economic classification criterion, found a more incomplete basic vaccination schedule in economic classes D and E, according to appropriate doses.^12^ In Indonesia, a study conducted in 2012, with 18,021 children, revealed economic factors associated with incomplete vaccination up to 12 months of age, such as belonging to the poorest class and the mother's low education.^7^

A systematic review of 23 studies discussed factors associated with non-adherence to the children's calendar between 0 and 24 months of age in 13 different countries. Among the factors most often associated with the incomplete vaccination schedule are the child's birth order, low maternal education and worse socioeconomic conditions.^26^

Among the possible factors associated with non-vaccination or delayed vaccination, it is worth noting the false impression that there is no longer a need for vaccination, the lack of knowledge of new generations regarding the importance of vaccination, fake news on social networks, the anti-vaccination movement, the parents’ fear of the post-vaccination reaction, the woman in the job market, the shortage of immunobiologicals and the fear of multiple simultaneous injections.^27^

As for the strengths of this study, we highlight the analysis of timely doses of vaccines and the complete schedule up to 12 months of age, unlike most studies, which, in general, consider only doses of vaccine applied in the population. Also, a strong point of this study is the information collected to analyze factors associated with the incomplete vaccination schedule and the use of ICE.

The probabilistic sampling used reduced the selection bias, and the information obtained directly from the vaccination booklet reduced the measurement bias.

A limitation of this study was the small number of children who received vaccines in the private network (n=6), which did not allow a comparison with children vaccinated in the public network. Other limitations consist of the lack of more recent information in relation to the context, as they come from the last Census of 2010, in the analysis of the causes of low coverage and in the vaccines lacking in the studied period.

This study made it possible to identify factors associated with timely non-vaccination and recommended actions directed to health professionals and caregivers of children, especially in households with a greater number of children, in addition to greater incentive to actively seek children with vaccines delayed by health unit.
